# Supplementary material for: Lysine myristoylation mediates long-term potentiation via membrane enrichment of synaptic plasticity effectors
Source: EMBO J. 2025 Jun 17;44(15):4196–221. doi: 10.1038/s44318-025-00484-3 (PMC12316903; doi:10.1038/s44318-025-00484-3)
Supplement: Supplementary file 1 — Appendix [file 44318_2025_484_MOESM1_ESM.pdf]

**Appendix for: Lysine myristoylation mediates long-term potentiation via membrane enrichment of synaptic plasticity effectors**

**Table of Contents**

Appendix Table 1: *N*-terminal myristoyl modified peptide MRM……………….…..….………2

Appendix Table 2: Free fatty acid FFAST MRM transitions……………...………….....…..…3

**Appendix Table 1: *N*-terminal myristoyl modified peptide multiple reaction monitoring (MRM) targets and transition parameters**

| Protein | Gene Name | Accession | Amino Acid Sequence | RT (min) | Transition 1 | Transition 2 | CE |
|---|---|---|---|---|---|---|---|
| ******Exogenous Standard****** | | | | | | | |
| NEM modified Fyn synthetic peptide | FYN-Mal | | Myr-GC[NEM]VQC[NEM]K | 13.5 | 549.3 / 602.3 | 549.3 / 830.4 | 25.9 |
| *****Endogenous Standards***** | | | | | | | |
| L-lactate dehydrogenase B chain | LDHB | P16125 | FIIPQIVK | 3.5 | 479.3 / 584.4 | 479.3 / 697.5 | 22.5 |
| *****Myristoylated Targets***** | | | | | | | |
| 5'-AMP-activated protein kinase β | AAKB2 | Q6PAM0 | Myr-GNTTSER | 10.1 | 487.8 / 492.2 | 487.8 / 391.2 | 22.9 |
| ADP-ribosylation factor 1 | ARF1 | P84078 | Myr-GNIFANLFK | 16.3 | 617.4 / 739.4 | 617.4 / 592.3 | 29.3 |
| ADP-ribosylation factor 3 | ARF3 | P61205 | Myr-GNIFGNLLK | 16.1 | 593.4 / 691.4 | 593.4 / 544.3 | 28.1 |
| A-kinase anchor protein 12 | AKA12 | Q9WTQ5 | Myr-GAGSSTEQR | 10.01 | 551.8 / 620.3 | 551.8 / 707.3 | 26.0 |
| Brain acid soluble protein 1 | BASP1 | Q91XV3 | Myr-GGKLSK | 8.7 | 400.3 / 532.3 | 400.3 / 589.4 | 18.6 |
| Calcineurin subunit B type 1 | CANB1 | Q63810 | Myr-GNEASYPLEMCSHFDADEIK | 10.3 | 841.7 / 846.4 | 841.7 / 971.4 | 38.7 |
| cAMP-dependent protein kinase-α | KAPCA | P05132 | Myr-GNAAAAK | 10.4 | 406.8 / 545.3 | 406.8 / 360.2 | 18.9 |
| Caytaxin | ATCAY | Q8BHE3 | Myr-GTTEATLR | 11.5 | 529.8 / 520.9 | 529.8 / 589.3 | 25.0 |
| Distal membrane-arm assembly complex protein 1 | DMAC1 | Q9CQ00 | Myr-GSSFSGSTEFSAPAPPTVSTAVPANPPAK | 11.3 | 990.5 / 694.4 | 990.5 / 723.9 | 45.5 |
| Golgi reassembly-stacking protein 2 | GORS2 | Q99JX3 | Myr-GSSQSVEIPGGGTEGYHVLR | 9.3 | 747.4 / 621.8 | NA | 32.4 |
| Guanine nucleotide-binding protein G(i) α2 | GNAI2 | P08752 | Myr-GCTVSAEDK | 10.8 | 588.8 / 549.3 | 588.8 / 749.4 | 27.9 |
| Guanine nucleotide-binding protein G(i) α3 | GNAI3 | Q9DC51 | Myr-GCTLSAEDK | 11.4 | 595.8 / 549.3 | 595.8 / 662.3 | 28.2 |
| Guanine nucleotide-binding protein G(o) α | GNAO | P18872 | Myr-GCTLSAEER | 11.5 | 616.8 / 591.3 | 616.8 / 704.4 | 29.2 |
| LanC-like protein 2 | LANC | Q9JJK2 | Myr-GETMSK | 11.33 | 431.8 / 595.3 | 431.8 / 396.4 | 20.2 |
| MARCKS-related protein | MRP | P28667 | Myr-GSQSSK | 9.6 | 402.2 / 536.3 | 402.2 / 321.2 | 18.7 |
| MICOS complex subunit Mic19 | MIC19 | Q9CRB9 | Myr-GGTASTR | 10.5 | 430.3 / 434.2 | 430.3 / 363.2 | 20.1 |
| MICOS complex subunit Mic25 | MIC25 | Q91VN4 | Myr-GSAESAEAR | 10.2 | 544.3 / 533.3 | 544.3 / 662.3 | 25.7 |
| Mitochondrial amidoxime reducing component 2 | MARC2 | Q922Q1 | Myr-GSSSSTALAR | 10.6 | 573.8 / 618.4 | 573.8 / 705.4 | 27.1 |
| Myristoylated alanine-rich C-kinase substrate | MARCS | P26645 | Myr-GAQFSK | 11.7 | 424.3 / 580.3 | 424.3 / 509.3 | 19.8 |
| NADH dehydrogenase 1β7 | NDUB7 | Q9CR61 | Myr-GAHLTR | 9.4 | 432.8 / 389.3 | 432.8 / 526.3 | 20.2 |
| Neuronal calcium sensor 1 | NCS1 | Q8BNY6 | Myr-GKSNSK | 11.7 | 415.8 / 563.3 | 415.8 / 370.0 | 19.4 |
| PHD finger protein 24 | PHF24 | Q80TL4 | Myr-GVLMSK | 14.7 | 422.8 / 577.3 | 422.8 / 405.3 | 19.7 |
| Protein bassoon | BSN | O88737 | Myr-GNEASLEGGAGEGPLPPGGSGLG PGPGAGKPPSALAGGGQLPVAGAAR | 9.4 | 1082.1 / 641.4 | 1082.1 / 795.4 | 52.1 |
| Protein phosphatase 1A | PPM1A | P49443 | Myr-GAFLDKPK | 11.9 | 543.4 / 600.4 | 543.4 / 487.3 | 25.6 |
| Protein phosphatase 1G | PPM1G | Q61074 | Myr-GAYLSQPNTVK | 12.5 | 694.4 / 773.4 | 694.4 / 558.3 | 33.0 |
| SRC kinase signallinginhibitor 1 | SRCN1 | Q9QWI6 | Myr-GNAPSQDPER | 9.9 | 640.8 / 828.4 | 640.8 / 401.2 | 30.4 |
| Tyrosine-protein kinase Fyn | FYN | P39688 | Myr-GCVQCK | 10.7 | 481.3 / 534.3 | 481.3 / 307.1 | 22.6 |

## Appendix Table 2: Free fatty acid FFAST MRM transitions

| | MRM (m/z) ions | | |
|---|---|---|---|
| **Fatty acyl chain** | **FFAST-124** | **FFAST-127** | **FFAST-138** |
| C6:0 | 222.1 / 124.1 | 225.1 / 127.1 | 236.1 / 138.1 |
| C8:0 | 250.2 / 124.1 | 253.2 / 127.1 | 264.2 / 138.1 |
| C10:0 | 278.2 / 124.1 | 281.2 / 127.1 | 292.2 / 138.1 |
| C12:0 | 306.2 / 124.1 | 309.2 / 127.1 | 320.1 / 138.1 |
| C14:0 | 334.3 / 124.1 | 337.3 / 127.1 | 348.1 / 138.1 |
| C16:0 | 362.2 / 124.1 | 365.2 / 127.1 | 376.2 / 138.1 |
| C18:0 | 390.2 / 124.1 | 393.2 / 127.1 | 404.1 / 138.1 |
| C20:0 | 418.3 / 124.1 | 421.3 / 127.1 | 432.1 / 138.1 |
| C22:0 | 446.2 / 124.1 | 449.2 / 127.1 | 460.2 / 138.1 |
| C24:0 | 474.4 / 124.1 | 477.4 / 127.1 | 488.4 / 138.1 |
| C16:1 | 360.2 / 124.1 | 363.2 / 127.1 | 374.2 / 138.1 |
| C18:1 | 388.3 / 124.1 | 391.3 / 127.1 | 402.3 / 138.1 |
| C18:2 | 386.2 / 124.1 | 389.2 / 127.1 | 400.2 / 138.1 |
| C18:3 | 384.2 / 124.1 | 387.2 / 127.1 | 398.2 / 138.1 |
| C20:4 | 410.2 / 124.1 | 413.2 / 127.1 | 424.2 / 138.1 |
| C20:5 | 408.4 / 124.1 | 411.4 / 127.1 | 422.4 / 138.1 |
| C22:1 | 444.3 / 124.1 | 444.3 / 124.1 | 458.3 / 138.1 |
| C22:6 | 434.2 / 124.1 | 437.2 / 127.1 | 448.2 / 138.1 |
| C24:1 | 472.4 / 124.1 | 475.4 / 127.1 | 486.4 / 138.1 |
