# Supplementary material for: Lysine myristoylation mediates long-term potentiation via membrane enrichment of synaptic plasticity effectors
Source: EMBO J. 2025 Jun 17;44(15):4196–221. doi: 10.1038/s44318-025-00484-3 (PMC12316903; doi:10.1038/s44318-025-00484-3)
Supplement: Supplementary file 6 — Expanded View Figures [file 44318_2025_484_MOESM6_ESM.pdf]

Expanded View Figures

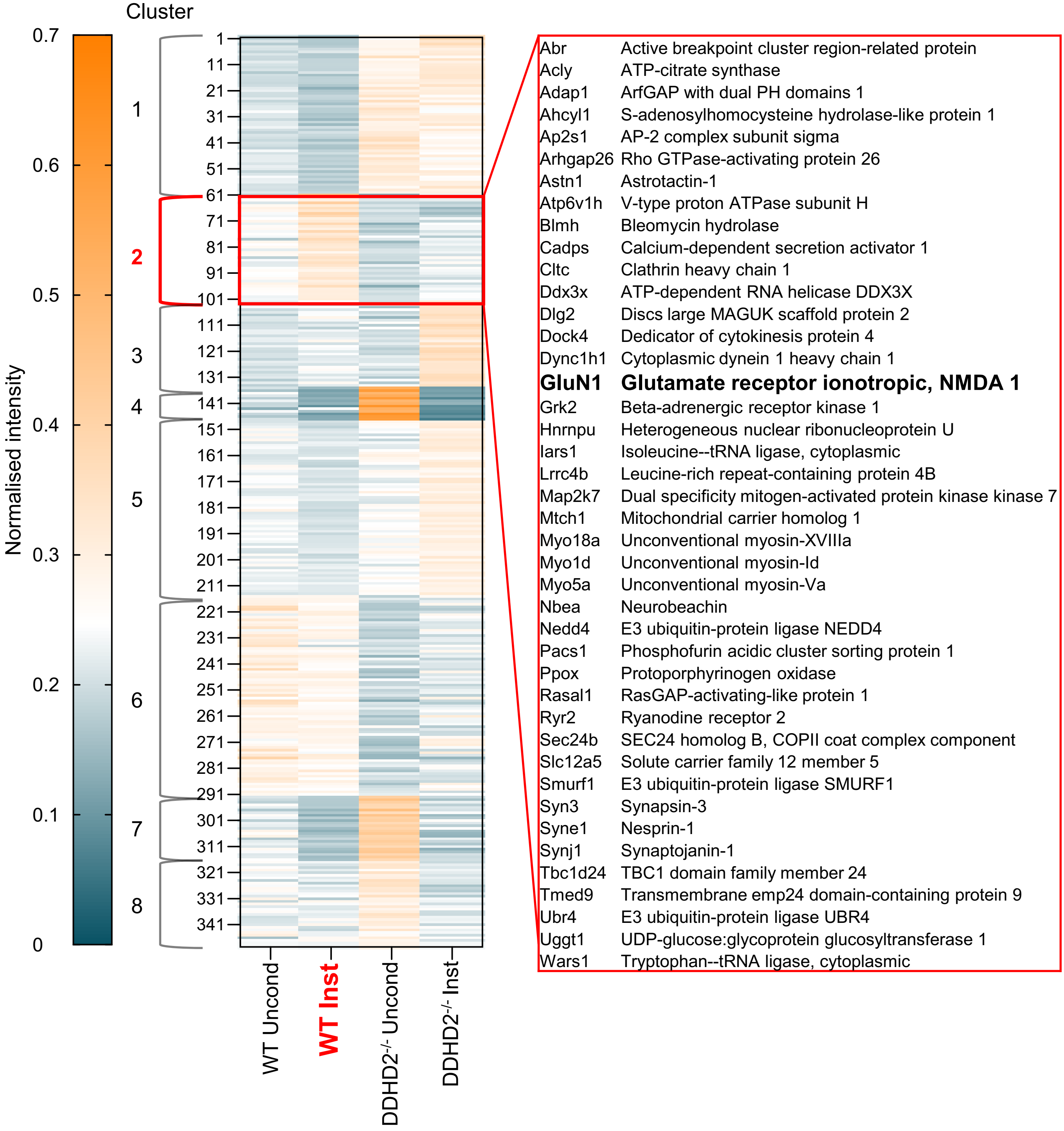

Figure EV1. Hierarchical clustered heatmap of the relative intensity of differentially expressed hippocampal proteins in instrumentally conditioned and control DDHD2$^{-/-}$ and C57BL/6 (wild type) animals.

Cluster 2 proteins display learning responsive increased expression, which is ablated in DDHD2$^{-/-}$ animals. Data Information: Differential protein expression was determined by one-way ANOVA, with p values ≤ 0.05 considered significant. Relative protein intensity was calculated by dividing the average intensity value for each condition by the sum of the average intensities for all conditions. Hierarchical clustering of relative protein intensities was performed using Ward's method.

© The Author(s)

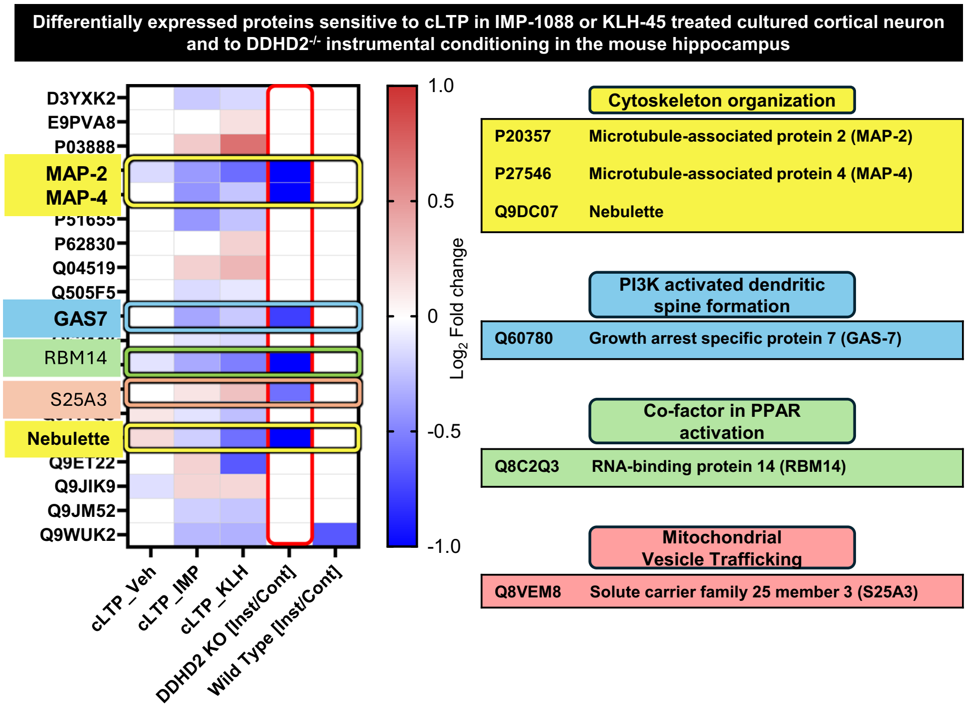

Figure EV2. Differentially expressed proteins sensitive to cLTP in IMP-1088 or KLH-45-treated cortical neurons and to DDHD2$^{-/-}$ instrumental conditioning in the mouse hippocampus.

Heatmap of the $\log_2$ fold change in proteins significantly changed (*t*-test *p* values ≤ 0.05) 2 h post-cLTP in vehicle (DMSO) and inhibitor (KLH-45 or IMP-1088) treated cortical neurons in vitro and instrumental conditioning in the hippocampus of DDHD2$^{-/-}$ and C57BL/6 wild type 12-month-old mice.

 © The Author(s)
